# Does continuing professional development enhance patient care? A survey of Irish based general practitioners: Successful implementation of mandatory CPD in Irish General Practice

**DOI:** 10.1186/s12909-022-03292-z

**Published:** 2022-03-31

**Authors:** Adam McBride, Claire Collins, Brian Osborne, Helen McVeigh

**Affiliations:** ICGP, 4-5 Lincoln Place, Dublin 2, Ireland

**Keywords:** General practice, Primary care, Continuing medical education, Patient-centred care, Quality improvement

## Abstract

**Introduction:**

The Irish Medical Council has regulated mandatory continuing professional development (CPD) for doctors since 2011 to enhance the quality and safety of Irish healthcare. The Irish College of General Practitioners (ICGP), as the professional body for general practitioners (GPs) in Ireland, operates a Professional Competence Scheme (PCS) for doctors working in general practice. As PCS evolves over time, it is important to measure the impact of mandatory CPD on patient care. The ICGP undertook this study to answer the research question: Does CPD enhance patient care? Research has been conducted on the impact of CPD on the medical profession, both in Ireland and abroad, on GP engagement with existing CPD supports and on the impact of CPD for GPs in other countries. To date, no study has been carried out in Ireland on GP views on the impact of mandatory CPD on patient care or on which type of CPD activity is perceived to be the most effective in this regard.

**Methods:**

All PCS enrollees on the 2018/2019 year who had provided an email address (*n* = 4,415) were asked to complete an anonymous online survey available in April and May 2019. The survey aimed to obtain feedback on existing CPD supports, enhancement of CPD supports, CPD impact on general practice and on patient care. The survey questions which related specifically to patient care were used to inform this paper.

**Results:**

A total of 1,233 (27.9%) PCS enrolees participated in the survey. Overall, 73.9% (*n* = 836) of respondents agreed that CPD assisted them in improving the quality of patient care with females significantly more likely to consider that CPD improved patient care. A total of 74.9% (*n* = 848) reported changes to patient management as a result of CPD activity and over half (56.4%; *n* = 464) of these believed that external CPD activity (courses/conferences) had the most potential to benefit their patient care, however, differences were observed across gender and age group.

**Conclusion:**

The majority of GPs who completed the survey found CPD engagement beneficial to their patient care. The majority of respondents agree that peer engagement activities are most likely to impact patient care thus demonstrating that mandatory CPD has been successfully implemented in this respect in Irish general practice. However, there is a difference in response to the various CPD formats across different demographic cohorts and this should be considered when designing the format of educational activities.

**Supplementary Information:**

The online version contains supplementary material available at 10.1186/s12909-022-03292-z.

## Introduction

While it was once universally accepted that all doctors engaged in professional development activities as a matter of course, changing healthcare needs have prompted many countries to introduce mandatory schemes as a means of encouraging and regulating this activity [1]. CPD Schemes focus on the continuing medical education (CME) and development of doctors when formal training is completed and encourage doctors to keep abreast of developments in their field of practice. Ireland introduced professional competence as a statutory requirement in 2011, regulated by the Irish Medical Council. All medical practitioners are obliged to enroll annually on a professional competence scheme (PCS) most relevant to their scope of practice. They are required to obtain and record a minimum of 50 CPD credits and one audit or practice improvement activity per year. Postgraduate Medical Training Bodies, including the Irish College of General Practitioners (ICGP), provide education, resources and online recording systems to facilitate doctors in their maintenance of professional competence. While the ICGP monitors CPD engagement through compliance metrics, this is not the only measure of success. Success can also be measured based on the value of CPD to doctors’ practice and patient care [2]. The ICGP developed this survey to seek GP input on answering the research question ‘Does CPD enhance patient care?’.

CPD combines CME with professional development and practice reflection to create a system focused on learning results benefitting both the patient and the wider community [3]. A previous review demonstrated that CME interventions could improve doctor performance and in some cases lead to better health care outcomes [4, 5]. However, while engaging in lifelong learning is a mandatory element of medical practice in many countries, learning does not automatically equate to enhanced patient care [6]. It is possible that this is due to didactic teaching methods being used to deliver education [7] or to doctors engaging in mandatory activity which they feel is not relevant to their patient care [8, 9]. It has also been noted that some doctors find the process more of a ‘tick box’ exercise than a way to enhance patient care [1]. It is possible that a gap between learning and practice is not always bridged through learning alone. It is worth noting that CPD surveys often measure a narrow range of impacts, focusing predominantly on knowledge, skills, attitudes and confidence [2]. Previous studies have demonstrated that activities which emphasise participant and peer engagement tend to have a higher impact on professional practice [7, 10]. Furthermore, CPD delivered through multiple media is more likely to have an impact on learning and care [5, 11]. Danish GPs noted patient needs as their top priority when engaging in CPD activities [12], while a survey of Canadian GPs reported that they found the Practice Support Programme beneficial to patient care [13].

A previous Irish study examining the provision of CPD activity through a CME small group learning model found that 97.1% of participants attended CME small group learning in order to improve their clinical practice. The majority, 86.3%, of respondents confirmed they had changed their practice as a result of their engagement with small group learning [14]. The small group learning model combines learning with peer engagement, including practice reflection. Evidence demonstrates that this environment is more likely to impact clinical and patient care, including an increase in vaccine uptake, knowledge of the needs of specific patient groups and enhanced prescribing practices [14]. According to Maher et al., it is important to encourage doctors to engage in CPD that is relevant to their own scope of practice [9]. For example, some GPs may need to engage in a variety of CPD activities that encompass the breadth of their practice, including skills such as obstetric medicine, emergency medicine and minor surgery [2]. The central role of patient care was evident in a 2017 survey of GPs attending CME Small Group sessions [15].

Previous studies demonstrated the perceived impact of a particular type of GP CPD activity, CME Small Group learning, on patient care [14, 15] and on the attitudes and experience of all Irish registered medical practitioners regarding the impact of the PCS on doctors’ practice although this study did not specifically focus on GPs nor on patient care [16]. No research has been carried out specifically on how GPs consider CPD as a whole contributes to patient care.

In view of the evolving evidence regarding the purpose and efficacy of CPD on doctors’ lifelong learning and its impact on patient care, the ICGP undertook an online survey of all enrollees on the ICGP PCS in the 2018/2019 PCS year who had provided an email address. This anonymous survey is the first to examine the impact of mandatory CPD on GPs practicing in Ireland in terms of their own learning, practice and patient care. The aim of this paper is to explore if doctors working in Irish general practice consider that their engagement in CPD enhances patient care.

## Methods

### Setting

Doctors practicing medicine in Ireland are obliged to enroll annually on a PCS and to record a minimum of 50 CPD credits and one audit or practice improvement activity. This includes minimum requirements across four mandatory categories:

1. External CPD (maintenance of knowledge and skills through educational activities)
2. Internal CPD (self-declared practice reflection and development)
3. Personal learning CPD (self-declared learning relevant to scope of practice)
4. Clinical / Practice audit (quality improvement)

A fifth category, research and teaching, is desirable and not compulsory. ICGP provides supports, education and an online platform to assist GPs in meeting and recording these requirements.

### Participants

Doctors working 50% or more of their working week in Irish general practice are eligible to enroll on the ICGP PCS. The study population was all GPs enrolled on the PCS in the 2018/2019 year (1^st^ May 2018 to 30^th^ April 2019). Only those who supplied an email address on registration were sent the invitation email.

### Procedure

All doctors enrolled on the 2018/2019 ICGP PCS year (*n* = 4,448) and who had provided a contact email address (*n* = 4,415) were sent an invitation by email to participate in an anonymous online survey during April and May 2019. Doctors who completed the survey and read the suggested associated reading were advised that they could record their engagement for internal CPD credits where they felt this was appropriate, recognising that practice reflection is involved in response preparation. The email invitation included a participant information leaflet. One reminder was issued one week after the initial invitation and participants were given two months to respond. Participation was entirely voluntary. Ethical approval was obtained from the ICGP Research Ethics Committee; consent was obtained from participants and no identifying information or IP addresses were collected.

### Survey design

A cross-sectional online survey was undertaken. The survey aimed to examine GP attitudes to CPD and to identify any trends arising; the focus of this paper is on GP perspectives on the impact of CPD on patient care. The full questionnaire is included in the supplementary material (S1).

We undertook this survey following the Medical Council’s 2017 proposal to review the existing PCS. The ICGP identified a gap in research specifically for GPs regarding their engagement with PCS. Survey questions were informed by existing PCS requirements and by changes proposed by the Medical Council. A pilot was conducted by obtaining feedback from GP members of the ICGP PCS Committee and the ICGP GP Clinical Leads. In addition, frequently asked questions from GPs to the PCS Helpdesk that indicated where CPD supports were most needed were reflected in the questionnaire. Questions aimed to understand how GPs perceived CPD impact on patient care, both in how they relate to patients and how they improved their practice. The questionnaire was included in the research ethics application.

### Data analysis

Data analysis was undertaken using SPSS V25 software, using descriptive analysis. For numerical data, means were used to summarise data and chi-square tests were used for categorical comparisons. Data from a free text question on the impact of CPD on patient care was examined under broad categories relevant to general practice and grouped into overarching themes accordingly.

## Results

A total of 1,233 PCS enrolees participated in the survey, a response rate of 27.9%. However, 1,164 provided responses to the questions relating to patient care and these are the subject of this paper. Of these, 26.5% (*n* = 309) reported being in a single-handed practice (with or without support staff); 54% (*n* = 624) of respondents were female and 50.1% (*n* = 583) were aged 40–59 years (Table 1).

**Table 1:** Age range of respondents

Age range	Number of respondents	Percentage of respondents
< = 39	316	27.1%
40 – 49	334	28.7%
50 – 59	249	21.4%
60 – 69	205	17.6%
70 +	60	5.2%

The majority of respondents, (70.2%; *n* = 817), were also members of the national professional body for general practitioners, the ICGP, and 63.0% (*n* = 728) were enrolled on the PCS since its inception in 2011.

**Fig. 1:**
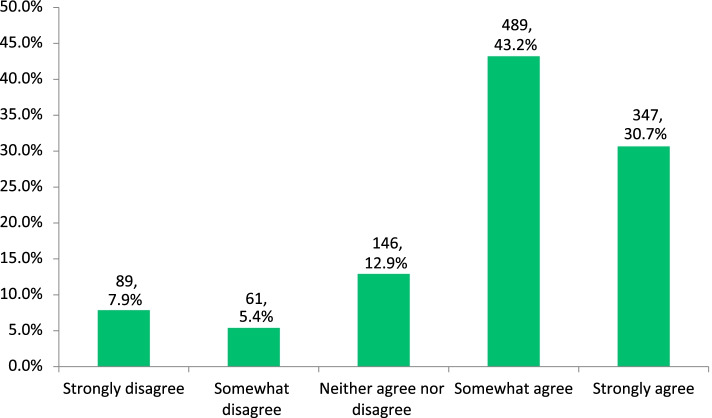
Impact of CPD on improving patient care

Among respondents, 30.7% (*n* = 347) strongly agreed that CPD assisted them in improving the quality of patient care with an additional 43.2% (*n* = 489) reporting that they somewhat agreed with this statement (Fig. 1).

Agreement that the scheme improved the quality of patient care was not significantly related to age group, working in single-handed or group practice, whether enrolled on the scheme from its inception or not or to membership of the ICGP. However, agreement that the scheme improved the quality of patient care was significantly related to gender with 76.5% of females agreeing with the statement compared to 70.7% of males (*p* =  < 0.05) (Table 2).

**Table 2:** Demographic variation on whether CPD improves patient care

**Demographic**	Agree (Strongly/Somewhat) % (n)	Disagree / Neither Agree nor Disagree % (n)	*p*-value
**Age**			*p* = 0.080
< = 39	71.2% (222)	28.8% (90)	
40 – 49	73.6% (237)	26.4% (85)	
50 – 59	70.9% (173)	29.1% (71)	
60 – 69	81.4% (162)	18.6% (37)	
70 +	76.4% (42)	23.6% (13)	
Gender*			*p* = 0.030
Female	76.5% (469)	23.5% (144)	
Male	70.7% (367)	29.3% (152)	
**Practice type**			*p* = 0.647
Single handed practice	72.8% (220)	27.2% (82)	
Group practice	74.2% (616)	25.8% (214)	
**Professional Body membership**			*p* = 0.301
Yes	74.8% (593)	25.2%(200)	
No	71.7% (243)	28.3% (96)	
**Member of PCS scheme since inception**			*p* = 0.779
Yes	73.5% (525)	26.5% (189)	
No	74.4% (311)	25.6% (25.6)	

^*^Chi-square analysis showed significant relationship *p*-value < 0.05

**Fig. 2:**
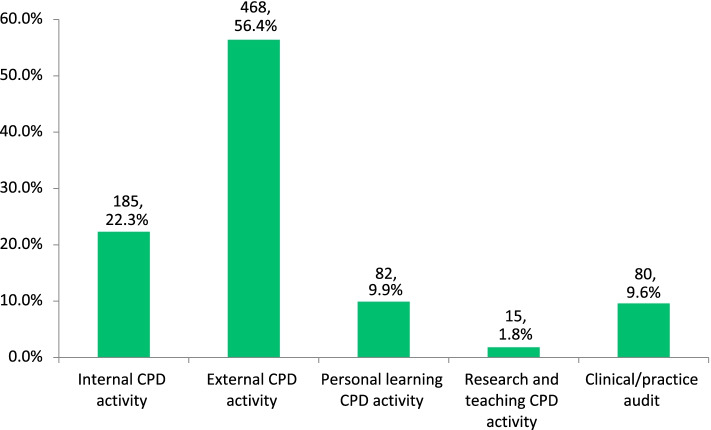
CPD activity category indicated by respondents to have the most potential to impact patient-centred care

Three quarters of respondents (74.9%, *n* = 848) reported that they have changed how they managed patients as a result of their CPD activity. Over half of these (56.4%, *n* = 468) consider that external CPD activity is most likely to contribute to changes in patient care with audit being the least likely of the mandatory categories to impact patient care (Fig. 2). Samples of external CPD enhancing patient care were provided by respondents and included engagement in CME Small Group meetings (*n* = 82) and attending courses or conferences (*n* = 50). Internal CPD was considered the second most likely category to impact patient care (22.3%, *n* = 185). GP respondents provided examples of internal CPD enhancing patient care; these included applying guidelines in practice (*n* = 126), enhanced record keeping (*n* = 44) and improved practice management (*n* = 26).

A significant relationship (*p* = 0.003) was observed between gender and the category of CPD considered to most impact on patient care. More females than males selected external CPD and audit, while more males selected internal CPD and personal learning (Table 3).

**Table 3:** Continuing professional development category of most impact on patient care by gender

CPD Category	Female % (n)	Male % (n)
External CPD	58.2% (266)	54.2% (202)
Internal CPD	19.7% (90)	25.5% (95)
Personal Learning CPD	7.9% (36)	12.3% (46)
Audit	12.5% (57)	6.2% (23)
Research & Teaching CPD	1.8% (8)	1.9% (7)

A significant (*p* < 0.001) relationship was also seen between age group and the category of CPD thought to impact on patient care with a higher proportion of younger age groups selecting external CPD and a higher proportion of older age groups choosing internal CPD (Table 4). This relationship between age and CPD category most impacting on patient care remains within each gender category with higher proportions of the younger age groups within both genders selecting external CPD compared to the older age groups.

**Table 4:** CPD category of most impact on patient care by age category

	**CPD Categories**				
Age	**External** **% (n)**	**Internal** **% (n)**	**Personal Learning % (n)**	**Audit** **% (n)**	**Research & Teaching % (n)**
≤ 39	65.0% (143)	11.8% (26)	9.1% (20)	12.3% (27)	1.8% (4)
40 – 49	59.2% (142)	22.1% (53)	11.7% (28)	5.8% (14)	1.3% (3)
50 – 59	46.1% (82)	30.3% (54)	10.7% (19)	10.1% (18)	2.8% (5)
≥ 60^a^	52.6% (101)	27.1% (52)	7.8% (15)	10.9% (21)	1.6% (3)

^**a**^ Due to small numbers, 60–69 and 70 + age groups combined

Survey respondents were asked to provide a free text description of how CPD impacted on how a patient was managed. Key areas referenced in these free text descriptions included prescribing (*n* = 171), chronic disease management (*n* = 164) and application of guidelines in practice (*n* = 126). Comments included the following:

- *“After engaging in education on COPD, when treating patients we began checking the patients' symptoms of breathlessness and history of exacerbations. For patients that continued to have exacerbations and for whom we were considering adding an inhaled corticosteroid (ICS) component to their inhalers we have been checking the patient's baseline eosinophil levels. The eosinophil levels have helped guide us on optimising the patients' inhalers by identifying which patients would be most suitable for ICS therapy.”*
- *“I have attended courses on menopause which was an area I felt needed development and as a result am much more confident safely prescribing HRT to women who need it.”*
- *“Our practice completed an audit on antibiotic prescribing using the anitbioticprescribing.ie guidelines. This resulted in a significant reduction in prescribing of ‘red’ antibiotics within our practice.”*
- *“Prior to a recent CPD meeting, I would have diagnosed Type 2 Diabetes Mellitus (DM) and commenced treatment with advice, referral to a dietitian, diabetic retinal screening and oral hypoglycemics (metformin). I now feel more confident adding in new medications.”*
- *“I was concerned that I was limiting STI testing to certain clinical presentations only. I did an audit of the relevant patient cohort including reviewing the STI guidelines. This improved my knowledge of the topic and made me more aware of the broader range of circumstances in which to carry out STI testing.”*
- *“Our practice carried out an audit on renal function monitoring in patients on NOAC therapy, previously we would have performed renal function on an ad-hoc basis, however on reviewing the guidelines that recommend 6 monthly to annual renal function and creatinine clearance in patients on NOAC this led us to introduce this as regular practice.”*

## Discussion

### Summary of findings

A response rate of 27.9% was achieved for the survey, which is in line with response rates from surveys of doctors internationally [15, 17]. The demographics of 1,164 respondents included in this paper are consistent with the overall PCS population – 54% of respondents were female while 54.7% of the full PCS population are female; 50.1% of respondents were aged 40–59 years, the comparative population data is 51.7% and 70% of respondents were also ICGP members compared to 72.8% overall.

The majority of survey respondents agreed that CPD has an impact on patient care with three quarters (74.9%) reporting that CPD has changed how they manage their patient care. Respondents were also asked to provide examples of the impact on patient care and these examples included improved prescribing, enhanced chronic disease management and a better knowledge of existing guidelines. More female than male GPs felt that CPD impacted patient care with 76.5% of females agreeing with the statement compared to 70.7% of males (*p* =  < 0.05).

Over half of respondents who reported a change in how they managed patient care (56.4%) believed that external CPD activity was most likely to benefit their patients’ care, whereas 22.3% indicated that internal CPD had the most impact on patient care. The type of CPD which respondents considered most impact their patient care varied with gender and age group.

### Interpretation in light of other findings/literature

Our findings show that GPs practicing in Ireland agree that their CPD activity is impactful and has improved the quality of patient care influencing how they manage patients. This is consistent with findings from previous studies that have shown continuing medical education improves doctor performance and patient health outcomes [5, 18]. Furthermore, where GPs found CPD impacted patient management, these findings are consistent with previous findings among GPs practicing in Ireland in relation to CME [15]. These findings also correlate with findings among Canadian GPs who reported being better able to care for their patients following engagement in the Practice Support Programme – a Canadian CPD programme [13].

A 2018 national survey examining all medical practitioners’ engagement with CPD in Ireland revealed that 40% of medical practitioners did not consider engagement with professional competence to be beneficial to patient care [16]. In contrast to this, only 25.1% of GPs surveyed here felt that CPD did not benefit their patient care. While 49% of responses to the 2018 national survey found that doctors do not map their CPD to their learning needs, findings from our survey suggest that GPs do seek and attend learning that matches their desire to enhance patient care. Differences between the two studies may reflect differences between GPs and doctors in other specialties and how they perceive the value of CPD.

### Perceived benefits of CPD

Of those who reported a change in how they managed patient care, 56.4% perceived external CPD activity – which includes educational events, conferences, lectures and CME small group learning – to be of most benefit to patient care. At the time of the survey, which was prior to the COVID-19 pandemic, the majority of external CPD activity in Ireland took place through face-to-face meetings that encourage peer engagement. That GPs find this type of activity beneficial to patient care reflects the findings related to CME study where 97.1% of respondents attended meetings to improve their clinical practice [14]. Previous literature suggests that didactic learning does not enhance patient care [7, 18], and it is likely that the peer engagement element of small group meetings is what GPs perceive as most beneficial to their practice [14].

Just over one quarter of those who reported a change in how they managed patients (22.3%) perceived internal CPD – activity encompassing practice reflection and development [2] – as being most beneficial to patient care. Engagement in internal CPD activity is self-declared by GPs when recording this activity in their online ePortfolios. In our study, the application of guidelines in practice was most often mentioned as a factor in improving patient care and this was reported elsewhere by Irish GPs as an ongoing CPD need [15].

### Sociodemographic responses

The type of CPD survey respondents considered most impacted their patient care varied with gender – 58.2% of female GPs felt that external CPD was most likely to benefit patient care, compared to 54.2% of their male counterparts. This aligns with findings from Australia where female doctors preferred professional development sessions and discussions – both external CPD activities – as a means of meeting CPD requirements [19]. Female GPs are more likely to work in part-time practice than their male peers, and this may make external CPD activities – not usually based within the practice – more accessible to them [20].

Our survey results demonstrated that 25.5% of male GPs felt that internal CPD was more likely to benefit patient care, compared to 19.7% of female GPs. This differs from the Australian report, which found that female doctors were more likely to engage in practice reflection (a key component of internal CPD) than male doctors [19].

Younger respondents to the ICGP survey were more likely to find external CPD beneficial to patient care – 65.0% of GPs aged ≤ 39 found external CPD most beneficial, compared to 52.6% of GPs ≥ 60 years. No comparable findings were located in the literature and as this relationship persisted within each gender, there is no confounding in that regard.

### Implications for research and practice

From regular member engagement and annual compliance figures, the ICGP is aware that the overwhelming majority of GPs practicing in Ireland are engaging in mandatory CPD activity. While most Irish-based GPs perceive particular CPD categories to be beneficial to patient care (e.g. external CPD), other GPs believe some categories of CPD (e.g. audit and internal CPD) are less beneficial for enhancing patient care. It is important to emphasise the correlation between patient care and individual CPD activities to underline the benefits of all CPD categories and to allow GPs to self-select the learning that most benefits their practice. Future research to establish the actual impact of CPD on patient care is warranted to assist GPs in selecting CPD that best matches their patient care needs. Actual impact could be investigated using metrics such as prescribing patterns, adherence to chronic disease management guidelines and audit outcomes. Equally important is the need to continue providing platforms for peer engagement to ensure that GPs maintain involvement in CPD activity that they find beneficial to their practice. The 2018 national survey found that the location of activities was a key barrier in meeting CPD requirements, so a method of integrating online activities with existing CPD supports may enhance the overall engagement with CPD [16]. Online activities, so common during the COVID-19 pandemic, may prove to be a way of encouraging peer engagement for those doctors who are less able to attend events in person for practice, public health or personal reasons. However, over time, online engagement may also reduce the opportunity for peer engagement – the impact of this in terms of benefit to patient care is unknown. The ICGP has already called for an assessment of the impact on patient care of a range of changes which have occurred due to the pandemic [21]. Further research on the impact of online learning and peer engagement would be useful in the development of CPD opportunities for the future.

### Strengths and limitations

This survey had some limitations as the sample of respondents was self-selecting. However, information was collected from a reasonably large sample of GP PCS participants from around the country, and the responding sample is broadly representative of the overall target population. The response rate of 27.9% may appear low but is typical of surveys of doctors internationally [15, 17]. The ICGP operates the only PCS for GPs in Ireland and this may have created a bias in responses, despite the anonymous nature of the survey. The ICGP encouraged study participation by advising GPs that they could record their participation for two internal CPD credits where appropriate. The survey was designed to encourage practice reflection when completing it, particularly for questions relating to impact on practice and patient care. It is possible that this incentive resulted in a disproportionate participation from GPs who habitually struggle to meet the annual internal CPD credit requirement.

## Conclusion

It is clear from this survey that the majority of GPs practicing in Ireland engage in CPD regularly and perceive this engagement to be beneficial to their patient care. CPD activities that encourage peer engagement and interaction are considered of most benefit to patient care by respondents. From the results of this survey, GPs practicing in Ireland appear to be adept at selecting CPD activity which suits their practice needs and at translating this into enhanced patient care. However, age and gender differences were observed in terms of the impact the various CPD activities on patient care and this should be considered when designing the format of educational activities.

## Supplementary Information

**Additional file 1.**

## Data Availability

The anonymous datasets used and / or analysed during the current study are available from the corresponding author on reasonable request.

## Abbreviations

CME: Continuing medical education
CPD: Continuing professional development
GPs: General practitioners
ICGP: Irish college of general practitioners
PCS: Professional competence scheme
