# Supplementary material for: Does continuing professional development enhance patient care? A survey of Irish based general practitioners: Successful implementation of mandatory CPD in Irish General Practice
Source: BMC Med Educ. 2022 Mar 31;22:220. doi: 10.1186/s12909-022-03292-z (PMC8969396; doi:10.1186/s12909-022-03292-z)
Supplement: Supplementary file 1 — Additional file 1. [file 12909_2022_3292_MOESM1_ESM.pdf]

Study to examine how the ICGP Professional Competence Scheme (PCS) enhances the qual
of general practice and patient care

Introduction

Before completing this survey, we advise that you review the information and guidelines on www.icgp.ie/pcs_survey.

Completion of this survey can be recorded for 2 internal CPD credits where you feel it is relevant to your practice evaluation and development.

The survey is in three parts; the 1st part covers demographics, the 2nd part relates to current PCS requirements and the 3rd part looks at potential developments and impact on patient care.

When responding to the questions in Part 1, please select the practice location where you have practised most regularly during the current PCS year (1st May 2018 – 30th April 2019).

If you wish to move back and forth through the survey questions, please use the ‘Prev' and 'Next' buttons at the bottom of the page and not the back button on your browser.

In order to proceed with the survey, please confirm the below:

* 1. I confirm that I have read the introductory information and email invitation for this survey.

☐ Yes

* 2. I understand that by completing this PCS survey anonymously, I consent to my data being used as part of this survey and any publications as a result of the findings.

☐ Yes

* 3. I understand that my data will be retained on a secure server and for a short period will be held on a server located outside the E.U. Data will only be held until the research is completed and it will then be deleted.

☐ Yes

Study to examine how the ICGP Professional Competence Scheme (PCS) enhances the qual of general practice and patient care

Part 1 - Demographic Information

* 4. Practice Location:

* 5. Population profile of this practice:

◯ Predominantly urban

◯ Predominantly rural

◯ Mixed urban and rural

* 6. What percentage of patients in this practice are GMS patients?

0% 100%

* 7. Please complete the following statement:
This practice is…

◯ a single-handed practice (with support staff)

◯ a single-handed practice (without support staff)

◯ a multi-GP practice

* 8. My age fits in the following range:

- ◯ <=29
- ◯ 30 - 39
- ◯ 40 - 49
- ◯ 50 - 59
- ◯ 60 - 69
- ◯ 70+

* 9. Are you a member of ICGP (please note: this refers to membership of ICGP which is separate from membership of the ICGP Professional Competence Scheme)?

- ◯ Yes
- ◯ No

Study to examine how the ICGP Professional Competence Scheme (PCS) enhances the qual of general practice and patient care

Demographic information

* 10. If you are a member of ICGP, have you accessed ICGP courses and/or Forum MCQs online during this PCS year?

- ◯ Yes
- ◯ No

Study to examine how the ICGP Professional Competence Scheme (PCS) enhances the qual
of general practice and patient care

Demographic information

* 11. If you are not a member of the ICGP, were you an ICGP member in the past?

- ○ Yes
- ○ No

Study to examine how the ICGP Professional Competence Scheme (PCS) enhances the qual
of general practice and patient care

Demographic Information

* 12. In Ireland, Professional Competence Schemes began in 2011. What year did you ***first*** join the ICGP Professional Competence Scheme?

- ○ 2011/12
- ○ 2012/13
- ○ 2013/14
- ○ 2014/15
- ○ 2015/16
- ○ 2016/17
- ○ 2017/18
- ○ 2018/19 (current year)

* 13. Are you semi-retired/retired?

- ◯ Yes, semi-retired
- ◯ Yes, fully retired
- ◯ No

* 14. In addition to your clinical work, do you work in any of the areas listed below? (Please choose all relevant options)

- ☐ Research
- ☐ Undergraduate education
- ☐ Postgraduate education
- ☐ None
- ☐ GP Training
- ☐ Other (please specify)

* 15. Please complete the following:
My clinical commitment is...

- ◯ 8 or more sessions per week
- ◯ 5 - 8 sessions per week
- ◯ 1 - 5 sessions per week
- ◯ Locum
- ◯ Sessional
- ◯ Not currently in clinical practice

* 16. What is your gender?

- ◯ Female
- ◯ Male

icgp

Study to examine how the ICGP Professional Competence Scheme (PCS) enhances the qual
of general practice and patient care

Part 2 - Professional Competence Scheme Requirements

All registered medical practitioners, regardless of working status, are required to meet the annual Professional Competence Scheme (PCS) requirements as set out by the Irish Medical Council.

These requirements are: 50 CPD credits (20 external, 20 internal, 5 personal learning and 5 additional credits in any of these categories or in research and teaching) and 1 Clinical/Practice audit per annum.

* 17. Does your internal CPD activity (practice evaluation and development) benefit you?

- ◯ Yes
- ◯ No

Study to examine how the ICGP Professional Competence Scheme (PCS) enhances the qual
of general practice and patient care

Professional Competence Scheme Requirements

* 18. If yes, in what way does your internal CPD activity benefit you?
(please choose all relevant options)

- ☐ It allows me to reflect on how I treat my patients
- ☐ It encourages me to discuss clinical cases with colleagues
- ☐ It encourages me to reflect on quality and patient safety in my practice
- ☐ Other (please specify)

Study to examine how the ICGP Professional Competence Scheme (PCS) enhances the qual
of general practice and patient care

Professional Competence Scheme Requirements

* 19. If no, what difficulties do you experience in obtaining internal CPD credits? (please choose all relevant options)

- [ ] I do not have the opportunity to engage in practice meetings and/or case discussions
- [ ] It takes time away from my patient care
- [ ] I do not feel that it encourages practice reflection
- [ ] Other (please specify)

Study to examine how the ICGP Professional Competence Scheme (PCS) enhances the qual
of general practice and patient care

Professional Competence Scheme requirements

* 20. Does your external CPD activity (maintenance of knowledge and skills) benefit you?

- ○ Yes
- ○ No

Study to examine how the ICGP Professional Competence Scheme (PCS) enhances the qual
of general practice and patient care

Professional Competence Scheme Requirements

* 21. If yes, in what way does your external CPD benefit you?
(please choose all relevant options)

- ☐ It gives me an opportunity to interact with colleagues to discuss practice and patient care
- ☐ It enhances my knowledge and skills for better patient care
- ☐ It keeps me up to date on quality and safety information
- ☐ Other (please specify)

Study to examine how the ICGP Professional Competence Scheme (PCS) enhances the qual
of general practice and patient care

Professional Competence Scheme Requirements

* 22. If no, what difficulties do you experience in obtaining external CPD credits? (please choose all relevant options)

- [ ] I find it difficult to access educational activity due to my working circumstances
- [ ] I cannot find activity that interests me or that relates to my practice
- [ ] I provide cover for other GPs and this impacts my ability to attend educational activity
- [ ] Other (please specify)

Study to examine how the ICGP Professional Competence Scheme (PCS) enhances the qual
of general practice and patient care

Professional Competence Scheme Requirements

* 23. Does your personal learning CPD benefit you?

- ( ) Yes
- ( ) No

Study to examine how the ICGP Professional Competence Scheme (PCS) enhances the qual
of general practice and patient care

Professional Competence Scheme Requirements

24. If yes, in what way does your personal learning CPD benefit you?
(please choose all relevant options)

- [ ] It encourages me to keep up-to-date for my patients
- [ ] It expands my knowledge of patient care
- [ ] I enjoy reading journals/online articles
- [ ] Other (please specify)

Study to examine how the ICGP Professional Competence Scheme (PCS) enhances the qual
of general practice and patient care

Professional Competence Scheme Requirements

25. If no, what difficulties do you experience in obtaining personal learning CPD credits? (please choose all relevant options)

- [ ] I do not know what to record in this section
- [ ] I do not feel it benefits my patient care
- [ ] I often forget to record activity in this category
- [ ] Other (please specify)

Study to examine how the ICGP Professional Competence Scheme (PCS) enhances the qual of general practice and patient care

Professional Competence Scheme requirements

* 26. Does your clinical/practice audit benefit you?

- ( ) Yes
- ( ) No

Study to examine how the ICGP Professional Competence Scheme (PCS) enhances the qual
of general practice and patient care

Professional Competence Scheme Requirements

* 27. If yes, in what way does your clinical/practice audit benefit you?
(please choose all relevant options)

- [ ] It encourages me to review the quality of my practice
- [ ] I have made changes to aspects of my practice as a result of an audit
- [ ] It gives me an opportunity to benchmark my practice against national/international guidelines
- [ ] Other (please specify)

Study to examine how the ICGP Professional Competence Scheme (PCS) enhances the qual
of general practice and patient care

Professional Competence Scheme Requirements

* 28. If no, what difficulties do you experience in meeting the annual audit requirement? (please choose all relevant options)

- [ ] I do not know how to carry out an audit
- [ ] I am unsure how best to audit my practice as my patient cohort changes regularly
- [ ] I find shorter quality initiatives to be more beneficial to my practice
- [ ] Other (please specify)

Study to examine how the ICGP Professional Competence Scheme (PCS) enhances the qual of general practice and patient care

Professional Competence Scheme Requirements

* 29. Generally, do you undertake activity in the research and teaching CPD category?

- ( ) Yes
- ( ) No

Study to examine how the ICGP Professional Competence Scheme (PCS) enhances the qual
of general practice and patient care

Professional Competence Scheme Requirements

* 30. If yes, on average how many hours of research/teaching do you engage in annually?

- ◯ 1 - 5 hours
- ◯ 5 - 10 hours
- ◯ 10 - 20 hours
- ◯ 20 - 40 hours
- ◯ 40+ hours

Study to examine how the ICGP Professional Competence Scheme (PCS) enhances the qual
of general practice and patient care

Part 3 - Potential for development and impact on patient care

* 31. I would prefer more flexibility between the internal, personal learning, research and teaching CPD credit categories

◯ Strongly disagree ◯ Somewhat disagree ◯ Neither agree nor disagree ◯ Somewhat agree ◯ Strongly agree

* 32. My engagement with CPD activity as part of the Professional Competence Scheme has assisted me in improving the quality of patient care

◯ Strongly disagree ◯ Somewhat disagree ◯ Neither agree nor disagree ◯ Somewhat agree ◯ Strongly agree

* 33. Have you changed how you managed your patients as a result of your CPD activity?

- ◯ Yes
- ◯ No

Study to examine how the ICGP Professional Competence Scheme (PCS) enhances the qual
of general practice and patient care

Potential for development and impact on patient care

* 34. If your management of patients has changed as a result of your CPD activity, please provide an anonymous example of this. By providing an example, you are consenting to the anonymised scenario potentially being used for the purposes of demonstrating how CPD activity can improve patient care.

Study to examine how the ICGP Professional Competence Scheme (PCS) enhances the qual
of general practice and patient care

Potential for development and impact on patient care

* 35. Which category of CPD activity do you feel has the most***potential*** to improve patient-centred general practice?

- ◯ Internal CPD activity
- ◯ External CPD activity
- ◯ Personal learning CPD activity
- ◯ Research and teaching CPD activity
- ◯ Clinical/practice audit

* 36. If ICGP ran courses in the following non-clinical topics, which would be of interest to you?
(please choose all relevant options)

- ☐ Communication
- ☐ Medicolegal
- ☐ Medical ethics
- ☐ Medical IT and technology
- ☐ Negotiation and conflict resolution
- ☐ Teamwork
- ☐ Leadership and motivation
- ☐ Time management
- ☐ Record keeping/GDPR
- ☐ People management

* 37. If ICGP provided further supports for meeting your internal CPD requirement, which would be of interest to you?
(please choose all relevant options)

- ☐ Quick Reference Guides
- ☐ Meeting templates
- ☐ Sample internal activity
- ☐ Other (please specify)

* 38. If ICGP provided further supports for meeting your personal learning CPD requirement, which would be of interest to you?
(please choose all relevant options)

- ☐ Suggested reading lists (recommended by peers)
- ☐ Sample personal learning activity
- ☐ Links to journal articles
- ☐ Other (please specify)

* 39. If ICGP provided further supports for meeting your research and teaching CPD requirement, which would be of interest to you?
(please choose all relevant options)

- [ ] Further information on recording this activity for CPD
- [ ] Templates for recording this activity
- [ ] Sample research and teaching activities
- [ ] I don't engage in research or teaching activity
- [ ] Other (please specify)

* 40. Which of the following would be of benefit to you in meeting your annual clinical/practice audit requirement?
(please choose all relevant options)

- [ ] More sample audits similar to those provided by ICGP on the audit webpage
- [ ] Training on how to conduct an audit
- [ ] Mechanisms to extract relevant audit data electronically in your practice
- [ ] More tools similar to iPCRN where practice specific reports are provided

icgp

Study to examine how the ICGP Professional Competence Scheme (PCS) enhances the qual
of general practice and patient care

Final page

41. Do you have any other comments about the ICGP Professional Competence Scheme that you would like to include?
